# Artificial Intelligence for Blood Glucose Level Prediction in Type 1 Diabetes: Methods, Evaluation, and Emerging Advances

**DOI:** 10.3390/s26092675

**Published:** 2026-04-25

**Authors:** Heydar Khadem, Hoda Nemat, Jackie Elliott, Mohammed Benaissa

**Affiliations:** 1 Department of Electronic and Electrical Engineering, University of Sheffield, Sheffield S1 4DE, UK; hoda.nemat@icr.ac.uk (H.N.); m.benaissa@sheffield.ac.uk (M.B.); 2 Department of Oncology and Metabolism, University of Sheffield, Sheffield S10 2RX, UK; j.elliott@sheffield.ac.uk; 3 Sheffield Teaching Hospitals, Diabetes and Endocrine Centre, Northern General Hospital, Sheffield S5 7AU, UK

**Keywords:** artificial intelligence, blood glucose level, diabetes mellitus, time series forecasting

## Abstract

Blood glucose level (BGL) prediction, by providing early warnings regarding unsatisfactory glycaemic control and maximising the amount of time BGL remains in the target range, can contribute to minimising both acute and chronic complications related to diabetes. This paper aims to provide an overview of data-driven approaches for BGL prediction in type 1 diabetes mellitus (T1DM). This review summarises different aspects of developing and evaluating data-driven prediction models, including model strategy, model input, prediction horizon, and prediction performance. It also examines applications of recent artificial intelligence (AI) techniques, including deep learning, transfer learning, ensemble learning, and causal analysis in the management of T1DM. Recent studies indicate that machine learning approaches often outperform classical time-series forecasting models in BGL prediction, particularly when using multivariate inputs. These findings also highlight the potential of advanced AI methods to improve prediction accuracy. Moreover, applying appropriate statistical analyses is essential to enable valid comparisons between different BGL prediction models, especially given the considerable inter-individual variability among people with T1DM. The development of efficient methods for integrating affecting variables into BGL prediction requires further research. Given the promising performance of advanced AI techniques and the rapid growth of AI innovation, continued exploration of cutting-edge AI strategies will be crucial for further improving BGL prediction models.

## 1. Introduction

Diabetes mellitus, also known as diabetes, is a growing metabolic disorder associated with serious short- and long-term complications that significantly affect global health. The occurrence of these complications can be delayed or even eliminated with effective diabetes management. The primary goal of diabetes management is to maintain blood glucose levels (BGLs) within the normal range [1]. Among the different types of diabetes, type 1 diabetes mellitus (T1DM) requires lifelong insulin therapy and intensive self-management. The literature has consistently emphasised the importance of self-management in T1DM, especially in reducing complications associated with the disease.

Over the past decade, the emergence of artificial intelligence (AI) has revolutionised the prevention, diagnosis, and management of diabetes. AI supports patients through personalised feedback and continuous monitoring while assisting clinicians in decision-making and treatment optimisation. Through applications such as clinical decision support, risk stratification, and patient self-management tools, AI shows great promise in transforming diabetes care. One of the most impactful AI applications in diabetes management is BGL prediction. Accurate BGL prediction enables timely insulin delivery and dietary adjustments, thereby improving glycaemic control and reducing the risk of adverse events. Consequently, BGL prediction has become a key component of both open- and closed-loop glycaemic control systems.

Several studies have reviewed AI applications in different fields of diabetes research, including prediction, diagnosis, complications, and management [2,3,4,5,6,7,8]. More focused reviews have explored AI in T1DM management [9,10] and in BGL and hypoglycaemia prediction [11,12,13,14]. However, existing reviews present several limitations. Most primarily focus on traditional machine learning models, offering limited discussion on advanced AI methodologies, including deep learning, transfer learning, ensemble learning, and causal inference, which have recently demonstrated superior performance in BGL prediction. Furthermore, few reviews provide a structured comparison of model design components or address the statistical and methodological challenges that often affect model validity and comparability across studies.

To address these gaps, this review was conducted through a comprehensive literature search across multiple databases, including PubMed, IEEE Xplore, and Scopus, using keywords such as “blood glucose prediction,” “diabetes mellitus,” “time-series forecasting,” “machine learning,” “artificial intelligence,” and “deep learning.” Titles and abstracts were screened to exclude irrelevant studies, followed by full-text evaluation of potentially eligible articles. The review provides essential knowledge on various aspects of developing data-driven BGL prediction models, with particular emphasis on state-of-the-art AI methodologies.

The paper begins with an overview of diabetes care and the fundamental components of BGL prediction model design, including modelling strategies, input selection, prediction horizons, and performance evaluation. It then reviews the application of advanced AI approaches—such as deep learning, transfer learning, ensemble learning, and causal analysis—in the context of BGL prediction. Furthermore, it discusses key methodological challenges and outlines statistical analyses critical for rigorous model evaluation. By consolidating these insights, this review offers a comprehensive reference for researchers aiming to develop and validate AI-based BGL prediction models. Although the primary focus of this review is on data-driven prediction models rather than glucose-sensing hardware, measurement technology and study design strongly influence the characteristics of the data used for model development. For this reason, this review also briefly discusses how sensor modality and retrospective versus prospective evaluation affect the interpretation and clinical translation of BGL prediction studies.

The emphasis on T1DM is deliberate, since it represents the most intensively studied setting for blood glucose forecasting and the principal clinical context for insulin-dosing support and automated closed-loop glucose control.

## 2. Diabetes Overview

Diabetes is characterised by the lack of insulin secretion from the pancreas, insulin sensitivity of body cells, or a combination of both. There are genetic and environmental risk factors that contribute to the development of the disease [1,2].

### 2.1. Types of Diabetes

The main types of diabetes are generally categorised as type 1 diabetes (T1DM), type 2 diabetes (T2DM), and gestational diabetes mellitus (GDM). T1DM is characterised by an absolute insulin deficiency, caused by the destruction of pancreatic beta cells, responsible for the production of insulin. Hence, people with T1DM require insulin therapy for the management of their disease. T2DM is characterised by insulin resistance. In T2DM, the pancreas is capable of producing insulin; however, the body cells become resistant to the effect of insulin. Also over time, the pancreas may lose its ability to secrete enough insulin, so there is a relative deficiency. The medications that are given to T2DM patients improve the secretion or absorption of insulin. After many years of T2DM, some patients secrete so little insulin that they too need insulin therapy. In GDM, the interaction between insulin and pregnancy-related hormones results in insufficient insulin to meet the extra demands of insulin resistance during pregnancy. This condition usually resolves after pregnancy [1,15].

Although this review focuses on T1DM, AI-based glucose prediction is also relevant to T2DM and GDM. However, the modelling objectives and data characteristics differ across these conditions. In T1DM, prediction is closely linked to insulin dosing, rapid glycaemic excursions, and closed-loop glucose regulation, which is why it has become the dominant setting for BGL forecasting research. In T2DM, greater heterogeneity in endogenous insulin production, medication use, and comorbidities may alter both model inputs and prediction targets. In GDM, pregnancy-specific physiological changes and a shorter disease management window create distinct modelling challenges. These settings warrant dedicated investigation, but methods developed in T1DM may still provide a useful foundation for broader diabetes applications.

### 2.2. Diabetes Complications

Deviations from normal BGL in diabetes can result in different short-term and long-term complications. The main short-term diabetic complications include hypoglycaemia and hyperglycaemia, which refer to the occurrence of low and high BGL, respectively. In the short term, hypoglycaemia can cause confusion, accidents, sometimes loss of consciousness or seizures, and rarely can be fatal. The main long-term diabetic complications are caused by chronic hyperglycaemia and can be divided into microvascular and macrovascular complications. Microvascular complications are characterised by damage to small blood vessels, such as diabetic neuropathy, nephropathy, and retinopathy, leading to amputations, kidney failure, and blindness respectively. In comparison, macrovascular complications affect large blood vessels, such as coronary artery disease, peripheral artery disease, and strokes. The occurrence of these complications can be delayed or even prevented by effective management of the disease [16].

### 2.3. Glycaemic Control

The key role of diabetes management is to aim to keep BGL in the normal range. Unlike healthy individuals whose BGLs remain in the normal range, people with T1DM can experience high or low BGLs. Blood glucose concentration is normally reported in milligrams per decilitre (mg/dL) or millimoles per litre (mmol/L) units. Based on the value of blood glucose concentration, three glycaemic states are defined: hypoglycaemia, normoglycaemia, and hyperglycaemia. BGL normally should be in the range of 70 mg/dL and 180 mg/dL, which is also called normoglycaemia. Hypoglycaemia is a situation in which BGL is below 70 mg/dL. Hypoglycaemia can be classified into three levels: mild (54 mg/dL < BGL < 70 mg/dL), moderate (40 mg/dL < BGL < 54 mg/dL), and severe (BGL < 40 mg/dL). Hyperglycaemia refers to a condition in which BGL is above 180 mg/dL. Hyperglycaemia can also be classified into three levels: mild (180 mg/dL < BGL < 250 mg/dL), moderate (250 mg/dL < BGL < 320 mg/dL), and severe (BGL > 320 mg/dL). The main limiting factor to achieve normoglycaemia is hypoglycaemia, which is associated with acute increased morbidity and mortality. So the primary challenge in diabetes management is the correction of hyperglycaemia without hypoglycaemia occurrence. To cope with this challenge, people with diabetes need to monitor their BGL frequently, and adjust medications accordingly [17,18].

### 2.4. Glycaemic Monitoring

There are three main glycaemic monitoring methods: HbA1c test as a long-term metric, self-monitoring of blood glucose (SMBG), and continuous glucose monitoring (CGM) sensors as short-term metrics [19]. The HbA1c test is a laboratory test that determines the percentage of HbA1c in the blood. This test provides an indicator of the average BGL during the past two to three months. It can be presented either in percentage (%) or millimoles per mole (mmol/mol) units. HbA1c is accepted as a gold standard marker for average glycaemic control; however, it does not measure glycaemic variability [1].

SMBG is a capillary measurement of BGL, providing a snapshot of the BGL at the time of measurement. Using glucometers, patients with diabetes need to prick their fingers to collect a blood sample. They then apply the sample on a test strip which is connected to a pocket-sized device. The device then identifies the glucose concentration using electrochemical, colourimetric, or optical procedures. SMBG can be used at all times of the day. Most patients are advised to perform SMBG four to eight times a day, specifically pre-meals, occasionally after-meals, bedtime, pre-exercise, and when there is a suspicion of hypoglycaemia. There is a positive association between the frequency of SMBG and glycaemic control improvement. This glycaemic monitoring method is comparably inexpensive and easy to learn; however, it is inconvenient and painful. Moreover, each measurement presents only a snapshot of BGLs, so it may miss glucose excursions [20,21].

To overcome the limitations of the SMBG method, CGM sensors have been introduced. They are portable devices that measure glucose concentration regularly and provide comprehensive glycaemic monitoring. Based on the fact that the glucose concentration of interstitial fluid (ISF) is similar to blood glucose concentration, CGM devices measure ISF glucose concentration. For this purpose, an electrode placed under the skin senses the glucose in the ISF and sends the signal wirelessly to an external apparatus which identifies the BGL. CGM sensors can continuously monitor BGL and assist people with diabetes to make more precise decisions about their glycaemic control. CGM also provides a variety of glycaemic factors such as time in glycaemic target, time in hypoglycaemia, glucose excursion, and intra- and inter-day glucose variability [21,22].

From a modelling perspective, measurement technology is not a neutral data source because it determines sampling density, signal continuity, calibration requirements, and error characteristics. SMBG provides sparse capillary blood measurements and is therefore useful for point-wise assessment but limited for continuous forecasting. In contrast, current T1DM BGL prediction research is dominated by minimally invasive CGM technologies, which provide dense time-series but measure interstitial rather than blood glucose and may therefore be affected by sensor-specific noise and physiological lag relative to vascular glucose [19,23]. Emerging optical, spectroscopic, and other non-invasive approaches have also been investigated as alternative routes for glucose-related monitoring [24,25,26,27]; however, such technologies are not yet represented in the mainstream public BGL prediction benchmarks reviewed in this paper.

## 3. Type 1 Diabetes Management

In T1DM, insulin therapy is required for the management of the disease and control of glycaemia. In T1DM management, glycaemic control can be affected by a number of factors. The main affecting factors include carbohydrate intake, physical activity (PA), and insulin dose [1].

### 3.1. Carbohydrate Intake

Meals greatly impact glycaemic control in T1DM patients. Food consumption affects BGL in a number of ways due to different physiological effects. In healthy individuals, absorbing carbohydrates from foods results in a temporary increase in BGL. The increase is automatically detected by the pancreas, and insulin secretion starts to return the BGL to a fasting level. The challenge of food consumption for people with T1DM is how BGL can be lowered in the shortest time after glucose absorption from carbohydrates. Fats, fibres, and proteins in foods can delay, slow, or decrease the glucose absorption process. Several measures have been introduced to quantify the impact of food on BGL, including the glycaemic index and the glycaemic load. These measures should be considered by patients for their glycaemic control [18,28,29].

### 3.2. Physical Activity

Regular PA can improve insulin sensitivity and reduce the risk of cardiovascular disease. Depending on the type, form, intensity, and duration of exercise, PA can significantly affect BGL in patients with T1DM [30,31]. However, proper management of glycaemia during and after PA is challenging for both patients and clinicians. It is difficult to accurately adjust insulin and carbohydrate during and after PA, and any mistake may cause hypoglycaemia or hyperglycaemia [32].

### 3.3. Insulin Therapy

Individuals with T1DM depend on injected external insulin to compensate for the lack of insulin secretion in the body. In general, two types of insulin are used: basal insulin and bolus insulin. Basal, as the background insulin, controls BGL in between meals and overnight, whereas bolus is used to manage BGL with meals. T1DM patients are required to consider the effect of their carbohydrate intake and PA levels when adjusting bolus doses to control their BGL, aiming for the normoglycaemic range [33,34].

Various insulin therapy solutions are available, including multiple daily injections, insulin pumps, and artificial pancreas (AP) systems. In multiple daily injections, patients with T1DM regulate their BGL by administering several injections of bolus and basal, daily. Insulin pumps, which administer insulin via an infusion cannula, are open-loop systems that require patients to adjust insulin dosage manually [35,36]. An AP is a closed-loop glucose control system that mimics the function of a healthy pancreas to regulate BGL for T1DM patients. An AP system typically consists of a CGM sensor, an insulin pump, and a controlling algorithm to adjust the insulin dose based on the CGM information. The primary objective of an AP system is to determine the optimal insulin dose to maintain BGL in the normal range and to avoid occurrences of adverse glycaemic events, including hypoglycaemia or hyperglycaemia [18]. However, due to the subcutaneous nature of insulin delivery, current systems have a significant time lag compared to healthy individuals, and the amount of carbohydrates consumed needs to be manually announced.

## 4. Artificial Intelligence

In general, intelligence is described as a set of capabilities, including analysing, learning, and reasoning, that can be used for solving problems and making decisions. AI is a field of computer science that aims to replicate these capabilities computationally and simulate human intelligence to analyse information and make sophisticated inferences. Main AI approaches can be categorised into three groups based on their objectives: learning from knowledge, reasoning from knowledge, and discovery of knowledge [3,37].

In healthcare, AI applications, combined with advances in medical devices and sensor technologies, have been increasingly used to extract insights from complex physiological data. This integration has enabled improved diagnosis, monitoring, and management of chronic diseases such as diabetes. In diabetes care, AI benefits patients, clinicians, and healthcare systems alike. By providing personalised assistance, AI enables patients to be informed, have continuous monitoring, and be able to independently manage their diabetes. Additionally, healthcare professionals can utilise AI for decision-making support. More specifically, applications of AI in diabetes management include prediction and detection of adverse glycaemic events, advisory systems for calculating insulin bolus, and BGL prediction. BGL prediction in people with T1DM is one of the most widely used AI applications in diabetes management.

### 4.1. Machine Learning

Machine learning is a computer program that enables computational models to learn and improve from experience. Machine learning tasks can be categorised as supervised, unsupervised, or reinforcement learning. Machine learning has contributed to developing accurate, personalised, and adaptive prediction models for managing T1DM. In particular, supervised learning is widely used to predict BGL values based on labelled datasets containing past glucose and influencing variables [12,13].

### 4.2. Time-Series Forecasting

An important aspect of AI in analysing and predicting data is time-series forecasting. Time-series forecasting, using historical data, attempts to find underlying patterns in the data to anticipate future time-dependent events. According to the number of variables measured over time, a time-series forecasting can be univariate or multivariate. In univariate forecasting, past measurements or observations of a single variable are used to predict future values, while in multivariate forecasting, multiple time-series are employed to make predictions [38,39,40]. Time-series forecasting is fundamental to BGL prediction. These models aim to learn temporal dependencies from past glucose data—and potentially other influencing variables—to anticipate future glucose fluctuations by attempting to find underlying patterns.

## 5. Blood Glucose Level Prediction

Advancements in medical sensors have enabled automatic continuous personal data collection. Furthermore, the developments of mobile health applications utilising AI strategies have advanced the self-monitoring and management of diabetes. One of the most important diabetes-related applications is the BGL alarm, which is based on an accurate BGL prediction model. It is proven that predicting BGL is a promising tool for glycaemic control. A BGL alarm significantly contributes to glycaemic control by providing patients with risk alerts, which allow them to take corrective precautionary actions based on the predicted BGL. This involves taking glucose prompted by a hypoglycaemia alarm and injecting correction insulin boluses following a hyperglycaemia alarm [41].

**Figure 1:**
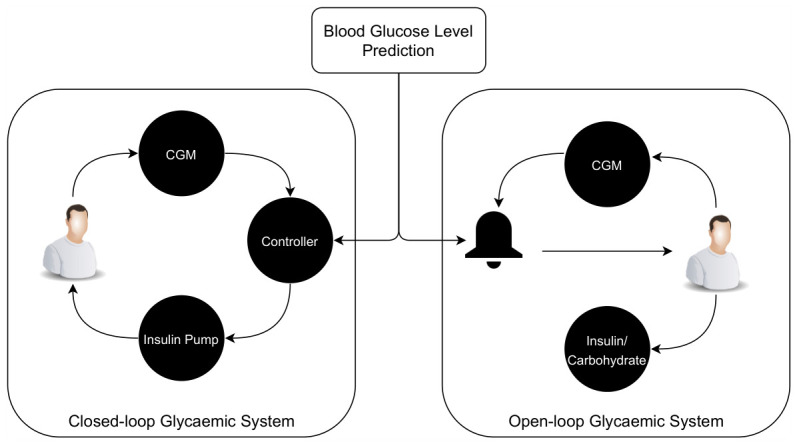
A schematic diagram illustrating the impact of blood glucose level prediction in both closed-loop and open-loop glycaemic systems on type 1 diabetes management.

Furthermore, estimating BGL in advance for a given prediction horizon is a key feature of closed-loop AP systems, which are the most advanced solution for T1DM management aimed at preventing adverse glycaemic events. Therefore, developing more reliable models for predicting BGL constitutes a critical AI-driven component of these systems [18,42,43]. Consequently, any improvement in BGL prediction has the potential to enhance diabetes management in both open-loop and closed-loop glycaemic control systems. Figure 1 illustrates the role and impact of BGL prediction in both types of systems for T1DM management.

The integration of prediction model strategies, input configurations, prediction horizons, and evaluation methodologies reveals the multifaceted nature of BGL prediction in T1DM management, which significantly influences model performance and practical utility. To ensure fair and rigorous evaluation across these diverse modelling frameworks, both regression-based and clinically oriented metrics—complemented by appropriate statistical analyses—are essential. Taken together, these elements highlight the importance of adopting a holistic and standardised framework for developing and assessing BGL prediction models that balance predictive accuracy with clinical applicability. The following subsections discuss these aspects in greater detail.

### 5.1. Prediction Model Strategies

Based on the model structure and knowledge requirements, BGL prediction algorithms can be classified into three main groups: physiological models (extensive knowledge), data-driven models (black-box approach), and hybrid models (intermediate knowledge) [9,23]. Among these approaches, the data-driven models have gained particular popularity, and increasing research has been devoted to exploring these approaches [11].

#### 5.1.1. Physiological Models

Physiological models aim to simulate the behaviour of real physiological systems using dynamic mathematical models. Hence, prior knowledge of physiological systems is a requirement for developing these models. These models are compartmental and derived by separating the body into uniform compartments. For BGL prediction, these models are designed to mathematically represent the dynamics of glucose-regulating systems. The glucose dynamics, the mechanism by which carbohydrate is converted to blood glucose, the process of insulin absorption, and the impacting model of PA on blood glucose regulation are the main compartments of a glucose–insulin physiological model for BGL prediction [9,18]. These models are not particularly precise, and the physiological constants must be specified depending on prior information on glucose-relevant factors [14,44].

#### 5.1.2. Data-Driven Models

Data-driven models, which use experimental data and pattern recognition techniques to simulate glucose dynamics, have been proposed to overcome physiological models’ limitations [45,46]. Data-driven, also known as empirical dynamic, models are black-box models generated from data only. These models, without any prior knowledge about the dynamics of glucose-regulating systems, by determining the relation between the past, present, and future BGLs, can provide accurate predictions of glucose dynamics. These models’ advantages include no need for physiological information, minimal user interaction, and ease of development [11,47]. Due to rapid advancements in data-driven AI methods, data-driven models have attracted considerable attention and are being increasingly explored [9,14]. These models could be mainly classified into classical time-series forecasting and machine learning approaches.

##### Classical Time-Series Forecasting

Classical time-series forecasting approaches have also been used for the BGL prediction task [48]. Autoregression (AR) [49], autoregressive moving average (ARMA) [50], autoregressive integrated moving average (ARIMA) [51], autoregression with exogenous variables (ARX) [52], autoregressive moving average with exogenous variables (ARMAX) [53], and autoregressive integrated moving average with exogenous variables (ARIMAX) [54] are the common approaches used for BGL prediction. In the first three models, it is presumed that the future BGL would be a linear function of the historical BGL data, whereas the second three models incorporate exogenous variables into the univariate counterpart models.

##### Machine Learning Algorithms

The use of machine learning algorithms for time-series forecasting has become increasingly popular in recent years [24,25,55,56,57]. Time-series forecasting can be restructured to supervised learning by converting data to a number of samples with input and output components. In this way, standard machine learning approaches can be used. A rolling window can be used to transform time-series data into samples with current and historical information as inputs and upcoming information as outputs [38]. In the literature, various machine learning algorithms have been developed for BGL prediction. Most of these algorithms include artificial neural networks (ANNs) [58,59], decision trees [60,61], kernel-based algorithms [43,62], and regression techniques [63,64]. According to reviews done by Mujahid et al. [13] and Woldaregay et al. [12], ANN models achieved the greatest number of data-driven approaches for BGL prediction in the literature.

#### 5.1.3. Hybrid Models

Hybrid models combine both physiological and data-driven models to develop a BGL prediction model. Physiological models are often used as inputs for data-driven models, and the data-driven model component captures the association between the output of the physiological models and future BGL. Models of glucose dynamics [65], insulin dynamics [43], glucose–insulin dynamics [66], and meal absorption dynamics [67] are the most common physiological model components for developing hybrid models [11,18].

### 5.2. Prediction Models Inputs

Data-driven models require accurate and large enough datasets. Data can be acquired from clinical trials or diabetes patient simulators. These models generally use information from historical BGL data, with or without other inputs.

#### 5.2.1. Data Origin

There are two types of data for developing BGL prediction models, real data from clinical trials and in silico data from diabetes patient simulators. Clinical datasets are the most commonly used type of data for BGL prediction [13], and more than half of them were collected in free-living situations [14].

The Ohio T1DM dataset [68,69], which supports reproducible benchmarking, is the most widely used publicly available clinical dataset. Also, DirecNet [70], in which different protocols and data have been collected from children and adolescents with T1DM, is the second most frequently used clinical dataset in the literature [14].

The OhioT1DM dataset comprises two collections of data obtained from a total of 12 individuals with T1DM. The first dataset, including six participants, was released for the first BGL Prediction Challenge in 2018 [68], and the second dataset, involving six additional participants, was released for the second Challenge in 2020 [69]. These datasets are often referred to in the literature as Ohio_2018 and Ohio_2020, respectively. The datasets provide multimodal data streams collected from physiological sensors, complemented by self-reported life events. All patients received insulin pump therapy using either a Medtronic MiniMed 530G or MiniMed 630G insulin pump with a Medtronic Enlite CGM sensor for BGL monitoring (Medtronic MiniMed, Inc., Northridge, CA, USA). Participants wore a Basis Peak fitness band in the 2018 dataset (Basis Science, Inc., San Francisco, CA, USA) and an Empatica Embrace band in the 2020 dataset (Empatica Inc., Cambridge, MA, USA). In addition, data contributors reported carbohydrate intake estimations, meal times, and lifestyle-related events such as sleep, work, and illness. Each dataset contains approximately eight weeks of data, with the last 10 days reserved for testing and the remaining period used for training.

Also, according to the review performed by Woldaregay et al. [12], AIDA [71] and UVa/Padova [72] were introduced as the two most used simulators for generating diabetes-related data. These simulators are usually preferred for evaluating the effectiveness of newly developed strategies for diabetes management prior to clinical research [13].

It should also be noted that most widely used public BGL prediction datasets are derived from commercial CGM and insulin delivery ecosystems. This has supported reproducible benchmarking, but it also means that the literature is shaped by the properties of specific sensing technologies, including sampling frequency, interstitial fluid-based readout, calibration procedures, and sensor noise. Consequently, considerably less is known about how well current prediction models transfer across alternative sensing modalities, particularly emerging non-invasive measurement technologies.

#### 5.2.2. Input Variables

Common inputs of BGL prediction models are the current and past information on BGL, carbohydrates, bolus insulin, and PA. It is worth noting that depending on the type of PA bands used, there are different kinds of PA data. These include heart rate, the magnitude of acceleration, step counts, galvanic skin response, skin temperature, electrocardiogram, and electronic health record [11,13,73]. Based on the review performed by Woldaregay et al. [12], the most commonly used group of variables used for BGL prediction is BGL, carbohydrate, and bolus insulin. The second most frequently used set of variables includes BGL, carbohydrate, bolus insulin, and PA. Using only BGL data ranked as the third most commonly used input.

BGL prediction from CGM data alone facilitates practical application in the real world; therefore, there is no need to acquire and process data from multiple sensors and modalities. Hence, some work used BGL data only for developing data-driven prediction models [58,74,75,76,77,78,79]. However, a BGL prediction model could be more reliable and accurate by considering other variables. Hence, others used BGL data along with other variables [59,80,81]. Investigating different inputs to find out if other variables can contribute to better prediction would be beneficial. Hence, some attempts have been made in this regard; however, no consensus has been reached.

A recent focused review by Lubasinski et al. further emphasised the importance of nutritional inputs in T1D blood glucose prediction, highlighting that carbohydrate intake remains the most commonly incorporated dietary feature, while broader nutrient composition and mixed-meal characteristics are still less consistently exploited [82].

According to Zecchin et al. [83], the addition of carbohydrate and bolus information to CGM data can improve the performance of predicting BGL between 30 and 120 min in advance using a neural network. According to Hameed et al. [84], adding additional carbohydrates and bolus data may add more perturbations and does not always improve prediction accuracy. Jeon et al. [85] examined the effects of 19 monitoring and physiological variables contained in the Ohio T1DM dataset. A total of 15 combinations of the variables were created by grouping them into four classes. According to their paper, using all feature classes might improve BGL prediction by avoiding lost information.

Heterogeneous sensing may further improve BGL prediction when auxiliary modalities provide complementary physiological information, such as wearable-derived physical activity, heart rate, sleep, or insulin delivery logs. However, integrating additional sensors does not automatically improve performance, since poorly aligned, sparse, or noisy modalities may introduce irrelevant variation and reduce model robustness [73,83,84]. Therefore, multimodal sensor fusion should be selective and physiology-informed, with careful attention paid to temporal alignment, signal quality, missingness, and feature relevance. In practice, heterogeneous sensors are most useful when they capture mechanisms that are not fully observable from CGM alone, rather than simply increasing the number of input channels.

### 5.3. Prediction Horizons

The prediction horizon refers to the time interval for which the model forecasts BGL in advance. Prediction horizons can be broadly classified into short-term (ranging from 15 to 60 min), medium-term (90 to 240 min), and long-term (360 min to one week) [13]. Short-term horizons are particularly useful for anticipating imminent hypoglycaemic or hyperglycaemic events, as they align with the physiological delay between insulin administration, carbohydrate intake, and measurable glucose responses, enabling timely intervention [86]. Medium-term horizons are especially relevant for supporting real-time decision-making in automated insulin delivery and advisory systems, where proactive adjustments are necessary to maintain glycaemic stability [87]. Long-term predictions offer value for behavioural planning and clinical insight but are more challenging due to uncertainty introduced by external, unmodelled factors such as meals, stress, and physical activity [42].

The choice of an appropriate prediction horizon represents a trade-off between predictive accuracy and clinical utility. In practice, most studies have focused on short-term horizons, particularly 15, 30, and 60 min predictions, which have demonstrated acceptable levels of accuracy and clinical relevance [12]. For instance, in the two well-known Ohio BGL Prediction Challenges, participants were asked to predict glucose values 30 and 60 min ahead, establishing these intervals as de facto benchmark horizons in the research community [58,85,88].

### 5.4. Prediction Performance Assessments

BGL prediction models should be properly evaluated. There are various evaluation criteria for assessing BGL prediction performance. Moreover, statistical analyses should be conducted to obtain a conclusive validation for comparing two or more prediction models for several data contributors.

#### 5.4.1. Evaluation Criteria

The performance of developed prediction models can be evaluated using complementary regression-based criteria (also known as empirical accuracy) and clinical criteria (also known as clinical accuracy). Regression-based criteria quantify the numerical agreement between predicted and reference glucose values, whereas clinical criteria assess whether prediction errors remain acceptable from a treatment and safety perspective. Accordingly, metric selection should be guided by the intended application of the model, since mathematically accurate predictions are not always clinically safe, and clinically acceptable predictions may not fully reflect overall numerical fit.

##### Regression-Based Evaluation

Primary metrics of regression-based evaluation to calculate the overall performance of developed BGL prediction models include root mean square error (RMSE), mean absolute error (MAE), and coefficient of determination ($R^{2}$), which are calculated as in Equations (1), (2), and (3), respectively.

(1) $$RMSE=\sqrt{\frac{\sum_{i=1}^{N}{\left(y_{i}-\hat{y_{i}}\right)}^{2}}{N}}$$

(2) $$MAE=\frac{\sum_{i=1}^{N}\left|y_{i}-\hat{y_{i}}\right|}{N}$$

(3) $$R^{2}=1-\frac{\sum_{i=1}^{N}{\left(y_{i}-\hat{y_{i}}\right)}^{2}}{\sum_{i=1}^{N}{\left(y_{i}-\bar{y}\right)}^{2}}$$

In Equations (1)–(3), *N*, $y_{i}$, and $\hat{y_{i}}$ represent the testing set size, the reference value, and the prediction value, respectively. Also, in Equation (3), $\bar{y}$ denotes the average of the reference values.

##### Clinical Evaluation

To gain an understanding of the clinical implications of BGL prediction models’ performance, error grid analyses and the Matthews correlation coefficient (MCC) have been frequently used. Error grid analysis, including Clarke error grid (CEG) [89], Parkes error grid (PEG) [90], and surveillance error grid (SEG) [91], analyse and visualise BGL predictions using the comparison with the BGL reference values. SEG, which assigns risk values to each predicted BGL, is the most recently developed analysis. Also, a surveillance error (SE), which has been defined as the mean of a bilinear interpolation of the SEG, is calculated as a unique score for each individual [58].

MCC, which is a classification metric, has been used to assess the effectiveness of the models to distinguish adverse glycaemic events from normoglycaemic events [92,93,94]. Considering an adverse glycaemic event as a positive class and a normoglycaemic event as a negative class, MCC is then calculated as in Equation (4).

(4) $$MCC=\frac{\left(TP\times TN)-(FP\times FN\right)}{\sqrt{\left(TP+FP)(TP+FN)(TN+FP)(TN+FN\right)}}$$

In this equation, TP and TN indicate the correctly predicted adverse and normoglycaemic events, respectively. Also, FP and FN are the incorrectly predicted adverse and normoglycaemic events, respectively.

##### Cross-Comparison of Evaluation Metrics

These metrics provide complementary rather than interchangeable information, and their selection should be aligned with the prediction objective. RMSE is more sensitive to large prediction errors because of the squared penalty and is therefore particularly informative when missed hypoglycaemic or hyperglycaemic excursions are of primary concern. MAE provides a more directly interpretable average absolute deviation and is less influenced by occasional extreme errors, making it suitable for reporting overall prediction accuracy and for comparing models across datasets with different error distributions. $R^{2}$ is useful for describing how well a model explains glucose variability, but it should not be used alone, since a model may achieve a reasonable $R^{2}$ while still producing clinically unacceptable point-wise errors. In contrast, error grid-based metrics are preferable when the aim is to assess clinical safety and decision support utility, because they evaluate whether prediction errors would lead to benign, unnecessary, or dangerous treatment actions [89,90,91]. Among these, SEG offers finer-grained risk interpretation and is therefore particularly useful when a continuous assessment of clinical risk is desired [91]. MCC is most appropriate when the prediction task is formulated as adverse event detection, such as identifying impending hypoglycaemia or hyperglycaemia, because it evaluates class discrimination while accounting for class imbalance [92,93,94]. Accordingly, studies focused on continuous glucose forecasting should preferably report RMSE and MAE together, ideally complemented by $R^{2}$, whereas clinically oriented alarm or decision support applications should additionally report an error grid metric and, when adverse event classification is involved, MCC.

#### 5.4.2. Statistical Analyses

##### Comparing Two Prediction Models

To compare a newly developed prediction model with an existing or baseline model, a non-parametric Wilcoxon signed-ranks test [95] is an appropriate test for comparing two approaches across multiple datasets where no assumption of normality is applied.

##### Comparing More than Two Prediction Models

To compare more than two different prediction models, firstly, a Friedman test [96], which is the non-parametric counterpart of the ANOVA test [97], is conducted. If there is a significant difference between at least two prediction models, a post hoc test such as the Nemenyi test [98] is then performed to determine which models are performed significantly differently in a pair-wise fashion. Also, when performing multiple comparisons, the Holm procedure [99] should be used for correcting the significance level. Additionally, for visualising the post hoc results, a critical difference (CD) diagram [100] has been used [74,101].

#### 5.4.3. Interpretability and Explainability

In addition to predictive accuracy and clinical safety, interpretability is increasingly important for the adoption of BGL prediction models in real clinical workflows. Post hoc explanation frameworks such as Local Interpretable Model-agnostic Explanations (LIME) and SHapley Additive exPlanations (SHAP) can help identify which recent glucose values, insulin doses, meal events, physical activity signals, or engineered temporal features most influenced a model’s output [102,103]. In the context of hypoglycaemia prediction, such analyses can clarify whether a warning is being driven by plausible physiological patterns—for example, falling recent glucose, recent bolus administration, or exercise-related effects—or by unstable model behaviour. However, explanation methods should be interpreted carefully in time-series models, since feature importance may depend on lag structure, local temporal context, and correlations between modalities. Future BGL prediction studies would therefore benefit from reporting not only whether hypoglycaemia is predicted correctly, but also why the model considered that prediction likely.

**Figure 2:**
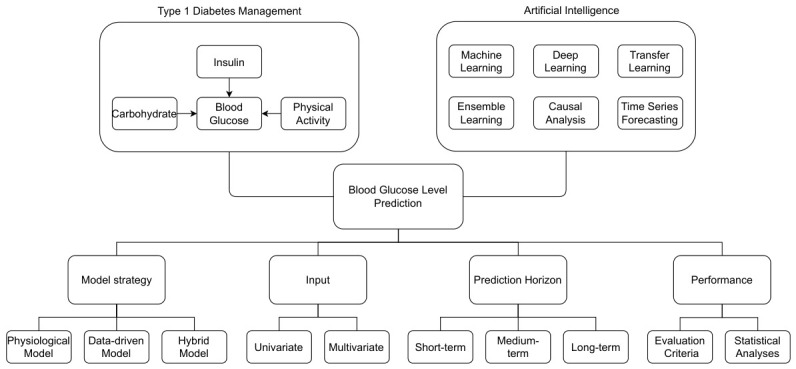
A schematic diagram for blood glucose level prediction.

Figure 2 illustrates a schematic diagram summarising the factors involved in the prediction of BGL.

## 6. Common Pitfalls

Several methodological issues recur in BGL prediction studies and can compromise the validity of reported results. One of the primary steps in developing BGL prediction models is handling missing and irregular data. This issue is not merely technical, since prior work has shown that data imputation choices can materially affect prediction performance [85]. A major concern is data leakage when imputed or interpolated values are introduced into the test set during model evaluation. In particular, interpolation within a missing test interval uses observations from both before and after the gap, thereby injecting future information into the evaluation window and leading to optimistically biased error estimates. Accordingly, the train/validation/test split should be created before any imputation, scaling, or feature engineering, and the reference targets used for validation and testing should always correspond to originally observed glucose values.

For short gaps in input variables, a practical strategy is to use only causal reconstruction methods based on past information, such as forward filling, last-observation-carried-forward for slowly varying variables, or restricted model-based extrapolation over a small maximum gap, while retaining the missingness mask and the time since the last observation as additional inputs [104]. For long or highly irregular gaps, simple extrapolation is often unreliable in T1DM because glucose dynamics are nonlinear and strongly affected by meals, insulin, and physical activity. In these situations, the preferred strategy is to segment the record into contiguous episodes, exclude prediction windows whose target or critical input history overlaps a long missing interval, and, when incomplete sequences must be retained, employ models designed for irregularly sampled or missing-aware time-series [104,105]. Reporting the percentage of excluded windows, the maximum tolerated gap, and whether missingness indicators were used can further improve transparency and reproducibility.

Another important issue is the distinction between retrospective and prospective evaluation. Most benchmark studies in BGL prediction are retrospective, meaning that models are developed and tested on previously collected datasets under offline conditions. Such studies are valuable for algorithm screening and controlled comparison, but they may overestimate practical performance because real-time deployment constraints, delayed or missing inputs, computation latency, alarm burden, and behavioural feedback are not fully represented. In contrast, prospective evaluation requires that only information available at the prediction time be used and that model performance be assessed in a forward-looking or real-time setting. This distinction is especially important when considering emerging non-invasive or less mature sensing technologies, where signal drift, calibration requirements, and temporal instability may differ substantially between offline analysis and real-world deployment. Therefore, future work should move beyond retrospective benchmarking toward more prospective and real-world validation frameworks whenever clinical translation is intended.

Another common problem is inadequate validation when comparing different prediction strategies. It should be noted that only a limited number of studies in the literature conducted thorough statistical analyses, and the majority of the studies compared average prediction performance across data providers. Comparing the averages of evaluation metrics across different datasets without accounting for their heterogeneity is not meaningful [100]. Since there is considerable inter-individual variability in the BGL patterns of people with T1DM [12], statistical analyses should be applied when comparing models to ensure that observed differences are significant and not due to random variation or dataset-specific effects.

## 7. Applications of Advanced AI Techniques

The literature shows an apparent acceleration in the utilisation of AI approaches for BGL prediction models. Improving the performance of BGL prediction is a challenging task, and even a small improvement is appreciated. Recently, the majority of studies have tried to incorporate new advanced AI strategies to investigate their capabilities to enhance BGL prediction performance. In this section, some emerging AI techniques applied in BGL prediction, including deep learning, transfer learning, ensemble learning, and causality analysis, are mentioned, and related research in the literature is discussed. Also, to provide some quantitative information regarding the performance of BGL prediction, the evaluation results of the top 10 methods introduced in the second BGL Prediction Challenge held in 2020 [106] are presented.

### 7.1. Deep Learning

Deep learning is a new area in AI which is inspired by the function of neurons inside human brains. In recent years, due to the growth of computing capability, deep learning models became more attractive.

Some of the most important network types are feed-forward neural networks (FNNs) [107], convolutional neural networks (CNNs) [108], recurrent neural networks (RNNs) [109], and more recently Transformer-based architectures [110]. FNNs are simple types of deep learning models which pass data in just one direction from the input layer to the output layer. These networks calculate the sum of the weighted inputs to find a mapping to output values. CNNs have convolutional layers. These types of layers consist of filters that are convolved with the input to extract local information from data [111]. RNNs are specially designed for time-dependent and sequence analyses. These networks have memory and feedback, and the output of a layer can be fed back to the input [112]. RNNs have a vanishing gradient problem in long data sequence analysis. To overcome this problem, LSTM [113] and gated recurrent units (GRUs) [114] were proposed. More recently, Transformer architectures and attention mechanisms have become highly influential in time-series forecasting because self-attention can model long-range temporal dependencies more directly than recurrent structures and can be trained efficiently in parallel [39,110]. Variants such as Informer and the Temporal Fusion Transformer (TFT) further extend this idea by improving scalability for long sequences and supporting interpretable multi-horizon forecasting [115,116].

Beyond recurrent and attention-based models, adversarial neural network frameworks have also emerged as promising architectures for BGL prediction. These include generative adversarial networks (GANs), which can improve forecasting by learning sharper glucose-trajectory distributions or supporting data augmentation under limited-data conditions [117]. More broadly, adversarial learning can also be used to reduce domain mismatch between simulated and real-world data or between heterogeneous patient cohorts, as illustrated by adversarial transfer learning approaches [118]. Most recently, Khadem et al. proposed a deep collaborative adversarial learning framework for personalised blood glucose forecasting in T1D and reported statistically significant improvements in both mathematical and clinical evaluation at 30 and 60 min prediction horizons on the Ohio datasets [119]. Although these methods remain less common than LSTM- and Transformer-based models in the current BGL literature, they are technically important because they address two persistent limitations of the field: small sample sizes and cross-domain variability.

Models based on deep learning may be more effective at detecting the dynamics of complex systems rather than traditional machine learning approaches and have shown promising results. In BGL prediction, where the ability to capture the physiological dynamics of glycaemia is vital for accurate prediction, different deep neural network configurations have been successfully developed [3,8,120]. Also, deep learning models have evolved into more advanced BGL prediction paradigms and have earned a distinct place in the 2018 and 2020 Ohio BGL Prediction Challenges [5,121]. In the BGL prediction context, attention mechanisms have already begun to influence model design. Early evidence includes neural attention-augmented LSTM models [122]. More recently, Transformer-based studies such as the Glucose Transformer have shown that self-attention can support both glucose forecasting and the prediction of impending hyperglycaemic and hypoglycaemic events [123]. Although Transformer-based BGL prediction is still less represented than LSTM- and ensemble-based approaches in the current literature, it constitutes an important emerging direction, particularly for leveraging longer temporal context, multivariate inputs, and more interpretable temporal dependency modelling [39,40]. The following provides an overview of some recent studies related to deep neural network models for BGL prediction.

Mirshekarian et al. [122] explored a number of experiments regarding the prediction of BGL based on data from CGM, insulin, meals, and activities. AIDA and UVa/Padova diabetes simulators were used as synthetic datasets, and the Ohio T1DM dataset was used as the real dataset. A memory-augmented LSTM model was developed for predicting BGL one hour in advance. An ARIMA model was also considered as a baseline, which was significantly outperformed by the LSTM model. According to the comparison results, the neural attention module enhanced the accuracy of prediction in synthetic data, but it did not make any improvement in real data. In contrast, adding daytime as an extra input to the LSTM model improved the performance of prediction in real data only.

Li et al. [92] developed a deep learning model using a convolutional RNN architecture for BGL prediction. The model was composed of convolutional and pooling layers, followed by a fully connected layer. They used BGL, bolus, and carbohydrate data from 10 virtual patients from the UVa/Padova simulator and 10 real people with T1DM as input. They evaluated and compared the proposed model with four baselines, including support vector regressions (SVR), an ARX, a latent variable model, and an ANN. The results showed the superior performance of the model compared to the baseline models.

Also, Martinsson et al. [58] developed a model using RNNs to predict BGL 30 and 60 min in advance without the requirement of preprocessing or feature engineering. Their end-to-end approach was developed and evaluated using BGL data of the Ohio T1DM dataset. A univariate Gaussian distribution was also used to estimate the certainty of the predictions. They evaluated their model using RMSE and SE metrics. Their method outperformed the naive baseline model.

Zhu et al. [80], employing dilated RNNs, developed a model that predicts BGL 30 min in advance. They investigated vanilla RNNs, LSTMs, and GRU architectures before selecting a vanilla RNN. In order to train their model, they used BGL, meal, and bolus data from the synthetic UVa/Padova and the real Ohio T1DM datasets. Their proposed model performed better in the synthetic dataset compared to the real one. Based on the fact that their model compared to AR, SVR, and CNNs performed better, they therefore concluded that the dilated RNN model would be more effective at predicting BGLs.

More recently, Alkanhel et al. developed a hybrid CNN-GRU model for multi-step-ahead blood glucose forecasting using a publicly accessible type 1 diabetes dataset and reported improved performance over standalone CNN, GRU, and LSTM models, highlighting the potential of lightweight hybrid deep learning architectures for real-time IoT-enabled diabetes management [124].

Likewise, Manchanda et al. showed that individual-specific LSTM models trained on limited subject-level data could achieve prediction accuracy and Clarke error grid performance comparable to aggregated training, supporting the feasibility of data-efficient personalised deep learning approaches for T1DM blood glucose forecasting [125].

### 7.2. Transfer Learning

In artificial intelligence, transfer learning refers to leveraging knowledge acquired in one source task, cohort, or domain to improve learning in a related target setting. Transfer learning involves adapting a pre-trained model to accomplish a related task more effectively. In BGL prediction, transfer learning mainly addresses three practical problems: the scarcity of subject-specific data required to train personalised models, the substantial inter-individual variability that limits direct model sharing across patients, and the domain shift between simulated and real-world glucose data. Accordingly, transfer learning can be used to initialise or adapt a model using data from other subjects, related diabetes cohorts, or simulator-generated datasets before fine-tuning it for an individual with sparse observations. This field has been successfully deployed in different areas, including computer vision, natural language processing, and healthcare [40,123,126,127]. Several studies have examined the efficacy of transfer learning in BGL prediction in recent years. Some of the recent work deploying transfer learning in BGL prediction is briefly described below.

Bhimireddy et al. [128] developed several sequence-to-sequence multivariate ANN architectures, including LSTM, BiLSTM, convolutional LSTMs, temporal convolutional networks, and sequence-to-sequence models for BGL prediction in T1DM. A gradient boosting algorithm was also used for selecting important features of the data in order to develop transfer learning models. They developed and evaluated their models using the Ohio T1DM dataset. Their results showed that sequence-to-sequence models outperformed transfer learning. Also, Zhu et al. [80] applied transfer learning to use the data of other patients for training each individual model. They discovered its effectiveness for a specific patient with various missing points.

Daniels et al. [126] investigated the effectiveness of multitask learning as a type of transfer learning in BGL prediction. They developed single-task learning, transfer learning, and multitask learning using a convolutional recurrent neural network. These approaches were compared with an SVR model, as a baseline model, and also with each other. They also considered BGL variability as proper knowledge for dividing the experiment into two groups to perform multitask learning. They used the OhioT1DM dataset for developing and validating their models. The results showed that the developed multitask learning approach outperformed other models for short-term and long-term prediction horizons. They concluded that multitask learning can be deployed for personalised models on limited individual data to promote BGL prediction.

De Bois et al. [118] developed a multi-source adversarial transfer learning architecture for enhancing data transfer quality between different sources. Their architecture allowed for the learning of a feature representation consistent across sources, making the learning process more universal and transferable. They utilised an SVR and two fully connected CNNs as baseline models. They also compared the proposed adversarial transfer models with standard transfer models. They used three different sources of data, including T1DM patients, T2DM patients, and a T1DM simulator. Their developed multi-source transfer learning could help with the lack of big enough data for training deep learning and improve the performance of BGL prediction.

Shuvo and Islam [129] incorporated multitask learning into a deep learning model to predict BGL in T1DM. Their proposed architecture was composed of two layers of stacked LSTM, as shared hidden layers, and two dense layers, as clustered hidden layers. These were followed by subject-specific dense layers. For developing their model, they used the Ohio T1DM dataset and evaluated it using RMSE, MAE, and CEG. The results showed an enhancement caused by multitask learning compared to other machine learning and deep learning models.

Taken together, these studies show that transfer learning in BGL prediction is best viewed as a data efficiency and personalisation strategy rather than a universal performance booster. The main problems it helps address are limited subject-specific training data, incomplete or sparse individual records, and weak generalisation across heterogeneous cohorts. A particularly important direction is simulator-to-patient transfer, where large in silico datasets can be used for pre-training and then adapted to real individuals, thereby reducing the amount of real labelled data required for personalisation [80,118,128]. This is especially attractive in T1DM, where obtaining large, well-annotated individual datasets is difficult. However, the benefit of transfer learning is not guaranteed; when the source and target domains are insufficiently aligned, direct sequence-to-sequence or subject-specific models may still perform better [128]. Therefore, the effectiveness of transfer learning depends on whether the transferred representation is well matched to the target patient and whether the adaptation is designed for cross-subject, cross-domain, or simulator-to-real transfer.

### 7.3. Ensemble Learning

Ensemble methods, as one of the advanced AI approaches, learn from several machine learning models, inferred as base-learners. The key idea of ensemble learning is that improvements can occur as a result of multiple base-learners compensating for the inaccuracy of a single base-learner. In addition to improving point-prediction accuracy, ensembles can also provide a practical indication of predictive uncertainty: agreement among base-learners suggests a more reliable forecast, whereas disagreement can flag low-confidence predictions that may require caution. Ensemble models are constructed in two main steps: generating base-learners and integrating base-learners. Considering base-learners’ generation, ensemble methods can be categorised as homogeneous and heterogeneous. In homogeneous ensembles, the base-learners are generated by a single algorithm, whereas in heterogeneous ensembles, at least two distinct algorithms are used. Base-learners’ combination, also inferred as output fusion, is the process of combining outputs from base-learners. The two main approaches for output fusion include weighting methods and meta-learning methods. Base-learners’ outputs can be weighted and averaged to make a single output [130,131]. Two levels of learning are involved in meta-learning algorithms. Multiple base-learners are trained at the first level, and their predictions are combined at the second level to make the final prediction. Bagging [132], boosting [133], and stacking [134] are three known meta-learning algorithms for constructing ensemble models.

Beyond improving average prediction accuracy, ensemble learning can outperform single-model approaches for two broader reasons. First, combining multiple learners can reduce the influence of measurement noise, self-reported meal inaccuracies, missingness artefacts, and other dataset-specific disturbances, because model-specific errors may be averaged out rather than amplified by a single predictor. Second, ensembles can improve generalisation across diverse individuals with T1DM by integrating complementary inductive biases, for example, linear and nonlinear learners, short-lag and long-lag models, or glucose-only and multivariate sub-models. This is particularly valuable in T1DM, where marked inter-individual variability in glucose responses often limits the robustness of any single modelling approach [12,131].

Since glucose dynamics are complex, ensemble techniques can be used to improve the accuracy of BGL prediction by combining multiple models [12,131]. Recently, several researchers have examined the use of different ensemble models for predicting BGL. Some of the recent work is briefly described below.

This reliability perspective is particularly relevant in T1DM management, where predictions near hypo- or hyperglycaemic thresholds can trigger high-stakes corrective actions. In such settings, an ensemble should ideally be assessed not only by its average prediction error but also by how informative the spread of its base-learner outputs is about model confidence. Related BGL studies have already demonstrated the value of explicit confidence or uncertainty estimation in neural forecasting [58,93,135]. Within ensemble learning, similar information can be extracted from the dispersion of base-learner predictions, for example, through their variance, range, or disagreement. This can support safer alarm generation, selective suppression of unreliable predictions, and stronger human trust in AI-assisted decision support.

In another study, Jeon et al. [85] investigated the prediction of BGL using the Ohio T1DM dataset over a prediction horizon of 30 min. Following their previous work [136], in which they showed that their developed gradient-boosted regression tree (XGBoost) model, consisting of an ensemble of decision trees with a gradient boosting framework, outperformed an LSTM model and a random forest regression, they developed five variants of the model using optimised parameters. To create an ensemble model, they further combined the predictions from five models using weighted output fusion models. They demonstrated that compared to the individual models, the ensemble model provided more accurate BGL predictions.

Saiti et al. [137] developed three ensemble models using ARX and SVR as base-learners, followed by linear, bagging, and boosting meta-learning output fusions. BGL, carbohydrate, and insulin were used for developing prediction models. The models were evaluated and compared for prediction horizons of 30, 45, and 60 min. Their results showed that the three ensemble models outperformed both individual learners.

In the Ohio BGL Prediction Challenge 2020, Nemat et al. [138] developed two methods leveraging ensemble learning for BGL prediction in T1DM using BGL and PA data. Three heterogeneous base-learners including a multilayer perceptron (MLP), an LSTM, and partial least squares regression (PLSR) were used. In one method, histories of BGL data appended with the average of PA in the same histories were used to train base-learners. In the other method, histories of BGL and PA were used separately to train the same base-regressions. The predictions from the base-learners were used as input for a PLSR model to create a combined model using a stacked meta-learner to make the final predictions. The results showed the efficacy of both methods for BGL prediction.

Also, in the same Challenge, using the Ohio T1DM dataset, Khadem et al. [139] developed ensemble models for BGL prediction utilising different lags. Three heterogeneous linear and ANN base-learners were trained using both 30 and 60 min of historical BGL data. These models were then used to create two uni-lag systems and one multi-lag system. In the uni-lag system, base-learners were trained using either 30 or 60 min of historical BGL data, whereas in the multi-lag system, base-learners were trained using both lags. In all three systems, a PLSR model was used as a meta-learner based on stacked meta-learning. The results showed that the stacking systems outperformed the individual models. Also, the multi-lag system achieved the best prediction accuracy.

Nemat et al. [74] proposed new architectures for predicting BGL in T1DM patients that leverage deep learning and ensemble learning. A vanilla LSTM network, a bidirectional LSTM network, and a linear regression model were used as three base-learners. The meta-learning output fusion strategy was then used to integrate base-learner outputs in three approaches including stacking, multivariate, and subsequences. The stacking method involved concatenating the base-learners’ outputs and feeding them into a linear meta-learner. As part of the multivariate approach, base-learners’ outputs served as multivariate input for training a multivariate LSTM model. The meta-learning thus considered univariate time-series forecasting as multivariate forecasting. The subsequences approach considered base-learners’ outputs as different subsequences to feed a convolutional LSTM. The impact of devised meta-learning strategies on the efficacy of BGL prediction was compared and benchmarked with non-ensemble models. The results showed that the proposed ensemble models statistically significantly outperformed the benchmarked non-ensemble models. Also, the two novel meta-learning approaches performed comparably to the effective stacked learning approach.

Also, Wadghiri et al. [131], in their review, verified that homogeneous ensembles, mostly followed by bagging and boosting meta-learning, improved the performance of BGL prediction compared to individual machine learning models, still comparable to deep learning models. Meanwhile, heterogeneous ensembles, mostly followed by stacking meta-learning, outperformed single machine learning and deep learning models. Hence, heterogeneous ensembles could be generally considered better than homogeneous ones in BGL prediction. Also, ANNs were determined as the most widely used base-learners for constructing heterogeneous ensembles, according to their review.

Furthermore, Khadem et al. [94] proposed a lag fusion framework employing meta-learning analysis to address the challenge of determining an appropriate history length for model training in the BGL prediction task. The developed method utilised MLP and LSTM models trained over 30, 60, 90, and 120 min of histories. Then, an interconnected lag fusion approach was developed based on nested ensemble learning. The analyses were performed using the Ohio T1DM dataset. The results showed that the proposed method was effective in BGL prediction.

Langarica et al. [140] proposed an ensemble model using meta-learning output fusion for personalised BGL prediction. They used synthetic data from the UVa/Padova simulator and showed that their model needed fewer data and training iterations compared to a transfer learning approach. Their model outperformed baselines, especially for longer prediction horizons.

Taken together, the advantage of ensemble learning in BGL prediction appears to arise not only from lower average error but also from noise mitigation, complementary representation learning, stronger cross-subject generalisation, and the possibility of uncertainty estimation from disagreement among base-learners. Heterogeneous ensembles are especially attractive because they combine models that respond differently to abrupt excursions, smoother trends, or auxiliary variables, thereby reducing dependence on a single modelling assumption [74,94,131]. Future work should therefore assess ensemble systems jointly in terms of accuracy, uncertainty, robustness to noisy inputs, and generalisation across heterogeneous individuals, since these properties are central to clinically reliable deployment.

### 7.4. Causal Analysis

In causal analysis, the relations between causes and effects are examined. Causal inference, as the main approach in causality analysis, quantifies causal relations [141,142]. Data-driven causal inference techniques have developed in recent years to discover causal associations between variables in time-series data [143]. These approaches assess causality from multivariate time-series based on observations of variables in complex systems [144,145]. Furthermore, causality has recently been applied in time-series forecasting tasks in several domains, such as neuroscience [146], climate [147], and economic data [148,149]. Using causality-based influencing features, these applications attempt to improve the performance of time-series forecasting.

In the literature, limited work used causal analysis in BGL prediction. Zhu et al. [150] developed a deep CNN as a modified version of WaveNet [151] for BGL prediction in people with T1DM. They categorised BGL prediction values into 256 classes; hence, the BGL prediction was converted from a regression task to a classification task. The classification model was mainly constructed using causal dilated CNN layers. They used BGL, insulin, carbohydrate, and the time index data from the Ohio T1DM dataset as input. Their results showed that their developed model differed from existing RNN models and compared to many current algorithms, it performed better.

He et al. [152] proposed a causal RNN for BGL prediction by mining the underlying causal links embedded in blood glucose. Their suggested system was made up of three primary modules, including an individual autocorrelation encoder, a learning module for personal attributes, and a CausalRNN module. They tried to model direct dependencies on BGL data and latent causal relations based on physiological parameters. The physiological features were extracted from carbohydrates of a meal, acting insulin amount, calorie expenditure, and sleep quality. They developed and evaluated models using a dataset from 112 people (38 with T1DM, 39 with T2DM, and 35 with no diabetes). Their results showed that their framework outperformed baseline models.

Also, Nemat et al. [101], as a pioneering work, investigated the feasibility of deploying causality information in order to improve the accuracy of BGL prediction in T1DM management. In the first phase of the investigation, the relations between BGL and carbohydrates, bolus, and PA were examined in the causality context. For this purpose, the causal relations were quantified using the convergent cross-mapping method [153]. Afterward, to determine the optimal lag of causality for each variable, the extended convergent cross-mapping method [154] was applied to quantify causality for various time lags. Next, two new approaches were proposed for utilising causality information as prior knowledge. In the first approach, causality strengths were used as the variables’ weights. In the second approach, the optimal causal lags and the corresponding causality strengths were considered as shifts and weights, respectively, for the variables. The performance of BGL prediction with and without leveraging causality was evaluated and compared using regression-based and clinical evaluation metrics and statistical analyses. Overall, the obtained results showed the effectiveness of developed causality-informed models in BGL prediction.

### 7.5. The BGL Prediction Challenge

The second BGL Prediction Challenge was held during the Fifth International Workshop on Knowledge Discovery in Healthcare Data in August 2020 [106]. Participants were tasked with predicting BGL values for two predefined prediction horizons of 30 and 60 min. It is worth noting that all participants used the Ohio_2020 dataset [69], which provided a common benchmark for evaluating model performance under consistent data conditions. The submitted models were evaluated using two primary error metrics, including RMSE and MAE, for both prediction horizons. The results were then compared and ranked to identify the most effective modelling strategies. Table 1 summarises the top 10 approaches, detailing their modelling methods and evaluation outcomes in developing BGL prediction models.

## 8. Benchmark of Prediction Models

Benchmark studies are particularly valuable because they move the literature beyond isolated model development and enable direct comparison of trade-offs between predictive accuracy, computational burden, interpretability, and clinical usefulness. However, because different studies use different datasets, input configurations, prediction horizons, and evaluation protocols, drawing general conclusions remains difficult. For clarity, Table 2 summarises representative benchmark studies in terms of their core algorithms, datasets, relative model complexity, interpretability, evaluation emphasis, and the main trade-offs identified.

Xie and Wang [88], for BGL prediction in T1DM, benchmarked a classical ARX model against 10 different machine learning approaches, including Elastic-Net, Lasso, Huber, Random-Forest, Gradient-Boosting-Trees, Ridge, SVR (linear and radial basis kernels), vanilla LSTM, and temporal convolution networks. They used the Ohio T1DM dataset and considered BGL, insulin, carbohydrate, and exercise data as input. Based on their results, in the 30 min prediction horizon, Ridge regression and ARX performed better. However, the ARX model overestimated peaks and underestimated valleys and had worse robustness than the DNNs.

Rodriguez et al. [162] developed and compared four machine learning approaches to support improved T1DM management: a Gaussian process with radial basis function kernels, an MLP, an SVM, and a Bayesian regularised neural network. They used glycaemia-related data, including BGL, meal, insulin injection, heart rate, step count, and sleep. The data was gathered under real-world conditions from 25 people with T1DM over a 14-day monitoring period as part of the Internet of Things. They showed that the Bayesian neural network performed best on $R^{2}$ and RMSE metrics and introduced it as the most capable technique for modelling BGL dynamics.

Moreover, Zhang et al. [120] compared four different data-driven models for BGL prediction in T1DM. The models included a dilated CNN model, a sequence-to-sequence LSTM model, a bidirectional reservoir computing model, and a newly developed multiple linear regression model. They used multiple variables measured by sensors or self-reported in the Ohio datasets. They found that while the sequence-to-sequence LSTM model had the best prediction performance for the 30 min prediction horizon, the multiple linear regression model performed the most accurately at BGL prediction for the prediction horizon of 60 min, with a lower computational cost.

Most recently, Nemat et al. [163] conducted a comprehensive benchmark analysis comparing the performance of different data-driven model structures for BGL prediction using both univariate and multivariate inputs. They compared the performance of various data-driven prediction approaches, including CTF, TML, and DNN, using three representative prediction models—ARIMA, SVR, and LSTM, respectively. The models were trained using BGL data as univariate input and were later extended to multivariate configurations incorporating carbohydrate, bolus, and PA data. Model performance was evaluated using both regression-based and clinically oriented metrics, followed by statistical analyses, including the Friedman test and a post hoc Nemenyi test, to enable valid comparisons. The analysis showed that, across more than half of the evaluated cases, considering various metrics, prediction horizons, and datasets, the three models performed comparably, particularly for univariate inputs. In the remaining cases, the SVR model demonstrated statistically significant superiority over at least one competing approach, while also being the fastest to train. Both the ARIMA and LSTM models exhibited similar performance patterns across all scenarios. Overall, the findings indicated that the SVR model provides the most effective balance of predictive accuracy and computational efficiency among the evaluated data-driven methods for BGL prediction.

Taken together, these benchmark studies suggest that no single modelling family is universally superior across all BGL prediction settings. Simpler or more classical models can remain highly competitive, especially for shorter prediction horizons, lower-complexity deployment settings, or when interpretability and computational efficiency are priorities. In contrast, more flexible deep or multivariate models may offer advantages when richer inputs and more complex temporal dependencies must be captured. Importantly, only the most recent comparative work has combined regression-based, clinically oriented, and statistical evaluation in a unified framework [163], highlighting the need for future benchmark studies to assess not only accuracy but also interpretability, robustness, and clinical relevance.

## 9. Conclusions and Future Directions

In this work, we conducted a comprehensive literature review to provide a concise guide for developing data-driven BGL prediction models for individuals with T1DM, with a particular focus on the use of advanced AI strategies. After presenting background information on diabetes care, key components involved in designing and evaluating BGL prediction models, including model strategy, input features, prediction horizon, and performance evaluation, were reviewed. Furthermore, the application of several state-of-the-art AI-based techniques, such as deep learning, transfer learning, ensemble learning, and causal analysis, in the context of BGL prediction were discussed.

Given the promising performance of advanced AI techniques reported in the literature, continued research into cutting-edge AI strategies for BGL prediction remains essential. In particular, ensemble learning approaches that combine multiple base-learners and employ diverse fusion mechanisms show potential to enhance model robustness and predictive accuracy. Beyond accuracy, future ensemble frameworks should also quantify predictive uncertainty from base-learner disagreement, since confidence-aware forecasts may be more suitable for trustworthy clinical decision support. Similarly, deeper exploration of causal inference methods, including the use of different causality detection approaches and the integration of causal lag information as prior knowledge, may improve both interpretability and predictive performance.

Another ongoing challenge lies in the selection and integration of input variables. Identifying the most informative features, including carbohydrate, insulin, and PA, and determining effective methods for incorporating them into predictive models, remains an open research problem.

Based on the literature reviewed, several pragmatic recommendations can be drawn. First, CGM-based prediction using routinely collected glucose, insulin, and meal-related information remains the most mature and clinically actionable paradigm for T1DM BGL forecasting. Second, when dataset size is limited and computational efficiency or interpretability are important, strong traditional baselines such as SVR should not be overlooked, since they can remain competitive with more complex models in benchmark comparisons [88,163]. Third, more complex deep learning, transfer learning, or ensemble approaches are best justified when richer multivariate inputs, longer temporal dependencies, or uncertainty-aware outputs can be exploited reliably. Finally, broader clinical translation will require evaluation across sensing technologies and more prospective real-world validation settings.

While this review has primarily focused on data-driven and deep learning-based approaches for blood glucose level prediction (BGLP), several other promising research directions remain underexplored and warrant attention in future work. These include exploring clinical trials that evaluate the real-world effectiveness of AI/ML-based BGLP models; exploring computational efficiency of models to make them suitable for edge computing devices or embedded chips; exploring mobile phone and wearable device implementations; and investigating the leverage of emerging generative AI techniques (e.g., GANs, transformers, and large language models) in BGL prediction. Integrating these aspects in future research could significantly enhance the practicality, transparency, and scalability of BGL prediction systems.

## Figures and Tables

**Table 1:** Comparison of the evaluation results of the second BGL Prediction Challenge.

Paper	Method	PH: 30 min		PH: 60 min	
		RMSE	MAE	RMSE	MAE
Freiburghaus et al. [155]	CRNN	17.45	11.22	33.67	23.25
Rubin-Falcone et al. [156]	DRNN	18.22	12.83	31.66	23.60
Hameed and Kleinberg [157]	VRNN	19.21	13.08	31.77	23.09
Zhu et al. [117]	GAN	18.34	13.37	32.21	24.20
Yang et al. [158]	MS-LSTM	19.05	13.50	32.03	23.83
Bevan and Coenen [159]	LSTM	18.23	14.37	31.10	25.75
Sun et al. [160]	LV	19.37	13.76	32.59	24.64
Khadem et al. [139]	MLS	19.01	13.73	33.37	24.98
Nemat et al. [138]	Stacking	18.99	13.73	33.39	25.04
Daniels et al. [161]	MTCRNN	19.79	13.62	33.73	24.54

Note: PH: prediction horizon; RMSE: root mean square error; MAE: mean absolute error; CRNN: convolutional recurrent neural network; DRNN: deep residual neural network; VRNN: vanilla recurrent neural network; GAN: generative adversarial network; MS-LSTM: multi-scale long short-term memory network; LSTM: long short-term memory; LV: latent variable, MLS: multi-lag stacking; MTCRNN: multitask approach using convolutional recurrent neural network.

**Table 2:** Summary of representative benchmark studies for blood glucose level prediction models.

Study	Core Algorithms	Dataset/Inputs	Complexity/Interpretability	Evaluation Emphasis	Key Findings and Trade-Off
Xie and Wang [88]	ARX, Elastic-Net, Lasso, Huber, Random-Forest, Gradient-Boosting-Trees, Ridge, SVR, LSTM, TCN	Ohio T1DM dataset; BGL, insulin, carbohydrate, and exercise	Mixed complexity; classical linear models more interpretable than DNNs	Primarily regression-based comparison; robustness discussed qualitatively; clinical metrics not emphasised	Ridge and ARX performed strongly at the 30-min horizon, but ARX overestimated peaks and underestimated valleys, whereas DNNs appeared more robust to extreme fluctuations.
Rodriguez et al. [162]	Gaussian process, MLP, SVM, Bayesian regularised neural network	25 free-living people with T1DM; BGL, meal, insulin injection, heart rate, step count, and sleep	Moderate-to-high complexity; neural models less interpretable than simpler statistical learners	RMSE and $R^{2}$ were highlighted; clinical metrics were not the main focus	The Bayesian regularised neural network performed best overall, suggesting benefit from richer multimodal input, although interpretability remained limited.
Zhang et al. [120]	Dilated CNN, sequence-to-sequence LSTM, bidirectional reservoir computing, multiple linear regression	Ohio datasets; multiple sensor-based and self-reported variables	Mixed complexity; multiple linear regression is the most interpretable and least computationally demanding	Performance comparison with explicit consideration of computational cost; clinical metrics were not emphasised	Sequence-to-sequence LSTM performed best at 30 min, whereas multiple linear regression performed best at 60 min with lower computational cost, highlighting an accuracy–cost trade-off.
Nemat et al. [163]	ARIMA, SVR, and LSTM representing classical time-series forecasting, traditional machine learning, and deep neural network structures	Univariate and multivariate settings incorporating BGL, carbohydrate, bolus, and PA	Broad classical-to-deep comparison; ARIMA offers higher interpretability, LSTM lower interpretability, and SVR an intermediate balance	Regression-based and clinically oriented metrics with Friedman and Nemenyi statistical analyses	The models were often comparable, but SVR offered the most consistent balance between prediction accuracy and computational efficiency across the evaluated scenarios.

Note: ARX: autoregression with exogenous variables; SVR: support vector regression; LSTM: long short-term memory; TCN: temporal convolutional network; MLP: multilayer perceptron; DNN: deep neural network; PA: physical activity. The complexity and interpretability descriptions are qualitative comparative summaries intended to highlight trade-offs rather than absolute rankings.

## Data Availability

No new data were created or analyzed in this study.

## References

[1] Association A.D. (2014). Diagnosis and classification of diabetes mellitus. Diabetes Care.

[2] Kavakiotis I., Tsave O., Salifoglou A., Maglaveras N., Vlahavas I., Chouvarda I. (2017). Machine learning and data mining methods in diabetes research. Comput. Struct. Biotechnol. J..

[3] Rigla M., García-Sáez G., Pons B., Hernando M.E. (2018). Artificial intelligence methodologies and their application to diabetes. J. Diabetes Sci. Technol..

[4] Contreras I., Vehi J. (2018). Artificial intelligence for diabetes management and decision support: Literature review. J. Med. Internet Res..

[5] Zhu T., Li K., Herrero P., Georgiou P. (2020). Deep learning for diabetes: A systematic review. IEEE J. Biomed. Health Inform..

[6] Ellahham S. (2020). Artificial intelligence: The future for diabetes care. Am. J. Med..

[7] Nomura A., Noguchi M., Kometani M., Furukawa K., Yoneda T. (2021). Artificial intelligence in current diabetes management and prediction. Curr. Diabetes Rep..

[8] Afsaneh E., Sharifdini A., Ghazzaghi H., Ghobadi M.Z. (2022). Recent applications of machine learning and deep learning models in the prediction, diagnosis, and management of diabetes: A comprehensive review. Diabetol. Metab. Syndr..

[9] Woldaregay A.Z., Årsand E., Botsis T., Albers D., Mamykina L., Hartvigsen G. (2019). Data-driven blood glucose pattern classification and anomalies detection: Machine-learning applications in type 1 diabetes. J. Med. Internet Res..

[10] Tyler N.S., Jacobs P.G. (2020). Artificial intelligence in decision support systems for type 1 diabetes. Sensors.

[11] Oviedo S., Vehí J., Calm R., Armengol J. (2017). A review of personalized blood glucose prediction strategies for T1DM patients. Int. J. Numer. Methods Biomed. Eng..

[12] Woldaregay A.Z., Årsand E., Walderhaug S., Albers D., Mamykina L., Botsis T., Hartvigsen G. (2019). Data-driven modeling and prediction of blood glucose dynamics: Machine learning applications in type 1 diabetes. Artif. Intell. Med..

[13] Mujahid O., Contreras I., Vehi J. (2021). Machine learning techniques for hypoglycemia prediction: Trends and challenges. Sensors.

[14] Felizardo V., Garcia N.M., Pombo N., Megdiche I. (2021). Data-based algorithms and models using diabetics real data for blood glucose and hypoglycaemia prediction–a systematic literature review. Artif. Intell. Med..

[15] Mellitus D. (2005). Diagnosis and classification of diabetes mellitus. Diabetes Care.

[16] Williams G., Pickup J.C. (2004). Handbook of Diabetes.

[17] Group I.H.S. (2017). Glucose concentrations of less than 3.0 mmol/l (54 mg/dl) should be reported in clinical trials: A joint position statement of the American Diabetes Association and the European Association for the Study of Diabetes. Diabetologia.

[18] Montaser E., Diez J.-L., Bondia J. (2020). Stochastic Seasonal Models for Glucose Prediction in Type 1 Diabetes. Ph.D. Thesis.

[19] Ajjan R., Slattery D., Wright E. (2019). Continuous glucose monitoring: A brief review for primary care practitioners. Adv. Ther..

[20] Khadem H. (2024). Advanced Artificial Intelligence and Machine Learning Driven Data Analyses in Diabetes Mellitus Research. Ph.D. Thesis.

[21] Beck J., Greenwood D.A., Blanton L., Bollinger S.T., Butcher M.K., Condon J.E., Cypress M., Faulkner P., Fischl A.H., Francis T. (2020). 2017 National standards for diabetes self-management education and support. Diabetes Educ..

[22] Wright L.A.C., Hirsch I.B. (2017). Metrics beyond hemoglobin A1C in diabetes management: Time in range, hypoglycemia, and other parameters. Diabetes Technol. Ther..

[23] Vettoretti M., Cappon G., Facchinetti A., Sparacino G. (2020). Advanced diabetes management using artificial intelligence and continuous glucose monitoring sensors. Sensors.

[24] Khadem H., Nemat H., Elliott J., Benaissa M. (2024). In Vitro Glucose Measurement from NIR and MIR Spectroscopy: Comprehensive Benchmark of Machine Learning and Filtering Chemometrics. Heliyon.

[25] Khadem H., Eissa M.R., Nemat H., Alrezj O., Benaissa M. (2020). Classification before regression for improving the accuracy of glucose quantification using absorption spectroscopy. Talanta.

[26] Gudiño Ochoa A., García-Rodríguez J.A., Cuevas-Chávez J.I., Ochoa-Ornelas R., Navarrete-Guzmán A., Vidrios-Serrano C., Sánchez-Arias D.A. (2024). Enhanced Diabetes Detection and Blood Glucose Prediction Using TinyML-Integrated E-Nose and Breath Analysis: A Novel Approach Combining Synthetic and Real-World Data. Bioengineering.

[27] Chellamani N., Albelwi S.A., Shanmuganathan M., Amirthalingam P., Alharbi E.M., Alatawi H.Q.S., Prabahar K., Aljabri J.B., Paul A. (2025). A Deep Sparse Capsule Network for Non-Invasive Blood Glucose Level Estimation Using a PPG Sensor. Sensors.

[28] Thomas D., Elliott E.J. (2009). Low glycaemic index, or low glycaemic load, diets for diabetes mellitus. Cochrane Database Syst. Rev..

[29] Schwingshackl L., Hobl L.P., Hoffmann G. (2015). Effects of low glycaemic index/low glycaemic load vs. high glycaemic index/high glycaemic load diets on overweight/obesity and associated risk factors in children and adolescents: A systematic review and meta-analysis. Nutr. J..

[30] Balkau B., Mhamdi L., Oppert J.M., Nolan J., Golay A., Porcellati F., Laakso M., Ferrannini E., Group E.R.S. (2008). Physical activity and insulin sensitivity: The RISC study. Diabetes.

[31] Tikkanen-Dolenc H., Wadén J., Forsblom C., Harjutsalo V., Thorn L.M., Saraheimo M., Elonen N., Rosengård-Bärlund M., Gordin D., Tikkanen H.O. (2017). Frequent and intensive physical activity reduces risk of cardiovascular events in type 1 diabetes. Diabetologia.

[32] Riddell M.C., Gallen I.W., Smart C.E., Taplin C.E., Adolfsson P., Lumb A.N., Kowalski A., Rabasa-Lhoret R., McCrimmon R.J., Hume C. (2017). Exercise management in type 1 diabetes: A consensus statement. Lancet Diabetes Endocrinol..

[33] Cefalu W.T., Leahy J.L. (2002). Insulin Therapy.

[34] Bazaev N., Pletenev A., Pozhar K. (2013). Classification of factors affecting blood glucose concentration dynamics. Biomed. Eng..

[35] Stephens E. (2015). Insulin therapy in type 1 diabetes. Med. Clin..

[36] Janež A., Guja C., Mitrakou A., Lalic N., Tankova T., Czupryniak L., Tabák A.G., Prazny M., Martinka E., Smircic-Duvnjak L. (2020). Insulin therapy in adults with type 1 diabetes mellitus: A narrative review. Diabetes Ther..

[37] Nilsson N.J. (2014). Principles of Artificial Intelligence.

[38] Brownlee J. (2018). Deep Learning for Time Series Forecasting: Predict the Future with MLPs, CNNs and LSTMs in Python.

[39] Lim B., Zohren S. (2021). Time-series forecasting with deep learning: A survey. Philos. Trans. R. Soc. A.

[40] Laursen R.A., Alo P. (2023). Transform Diabetes-Harnessing Transformer-Based Machine Learning and Layered Ensemble with Enhanced Training for Improved Glucose Prediction. Master’s Thesis.

[41] Nemat H. (2023). Artificial Intelligence in Blood Glucose Level Prediction for Type 1 Diabetes Management. Ph.D. Thesis.

[42] Oviedo Castillo S. (2019). Forecasting and Decision Support for Type 1 Diabetes Insulin Therapy Using Machine Learning. Ph.D. Thesis.

[43] Vehí J., Contreras I., Oviedo S., Biagi L., Bertachi A. (2020). Prediction and prevention of hypoglycaemic events in type-1 diabetic patients using machine learning. Health Inform. J..

[44] Hidalgo J.I., Colmenar J.M., Kronberger G., Winkler S.M., Garnica O., Lanchares J. (2017). Data based prediction of blood glucose concentrations using evolutionary methods. J. Med. Syst..

[45] Novara C., Pour N.M., Vincent T., Grassi G. (2015). A nonlinear blind identification approach to modeling of diabetic patients. IEEE Trans. Control Syst. Technol..

[46] Zarkogianni K., Mitsis K., Litsa E., Arredondo M.T., Fico G., Fioravanti A., Nikita K.S. (2015). Comparative assessment of glucose prediction models for patients with type 1 diabetes mellitus applying sensors for glucose and physical activity monitoring. Med. Biol. Eng. Comput..

[47] Sparacino G., Zanderigo F., Corazza S., Maran A., Facchinetti A., Cobelli C. (2007). Glucose concentration can be predicted ahead in time from continuous glucose monitoring sensor time-series. IEEE Trans. Biomed. Eng..

[48] Ståhl F., Johansson R. (2009). Diabetes mellitus modeling and short-term prediction based on blood glucose measurements. Math. Biosci..

[49] Gani A., Gribok A.V., Rajaraman S., Ward W.K., Reifman J. (2008). Predicting subcutaneous glucose concentration in humans: Data-driven glucose modeling. IEEE Trans. Biomed. Eng..

[50] Eren-Oruklu M., Cinar A., Quinn L., Smith D. (2009). Estimation of future glucose concentrations with subject-specific recursive linear models. Diabetes Technol. Ther..

[51] Yang J., Li L., Shi Y., Xie X. (2018). An ARIMA model with adaptive orders for predicting blood glucose concentrations and hypoglycemia. IEEE J. Biomed. Health Inform..

[52] Finan D.A., Zisser H., Jovanovic L., Bevier W.C., Seborg D.E. (2007). Practical issues in the identification of empirical models from simulated type 1 diabetes data. Diabetes Technol. Ther..

[53] Eren-Oruklu M., Cinar A., Rollins D.K., Quinn L. (2012). Adaptive system identification for estimating future glucose concentrations and hypoglycemia alarms. Automatica.

[54] Prendin F., Díez J.L., Del Favero S., Sparacino G., Facchinetti A., Bondia J. (2022). Assessment of Seasonal Stochastic Local Models for Glucose Prediction without Meal Size Information under Free-Living Conditions. Sensors.

[55] Khadem H., Nemat H., Elliott J., Benaissa M. (2022). Interpretable Machine Learning for Inpatient COVID-19 Mortality Risk Assessments: Diabetes Mellitus Exclusive Interplay. Sensors.

[56] Khadem H., Nemat H., Eissa M.R., Elliott J., Benaissa M. (2022). COVID-19 mortality risk assessments for individuals with and without diabetes mellitus: Machine learning models integrated with interpretation framework. Comput. Biol. Med..

[57] Khadem H., Nemat H., Elliott J., Benaissa M. (2022). Signal fragmentation based feature vector generation in a model agnostic framework with application to glucose quantification using absorption spectroscopy. Talanta.

[58] Martinsson J., Schliep A., Eliasson B., Mogren O. (2020). Blood glucose prediction with variance estimation using recurrent neural networks. J. Healthc. Inform. Res..

[59] Mirshekarian S., Bunescu R., Marling C., Schwartz F. (2017). Using LSTMs to learn physiological models of blood glucose behavior. Proceedings of the 2017 39th Annual International Conference of the IEEE Engineering in Medicine and Biology Society (EMBC).

[60] Seo W., Lee Y.B., Lee S., Jin S.M., Park S.M. (2019). A machine-learning approach to predict postprandial hypoglycemia. BMC Med. Inform. Decis. Mak..

[61] Calhoun P., Levine R.A., Fan J. (2021). Repeated measures random forests (RMRF): Identifying factors associated with nocturnal hypoglycemia. Biometrics.

[62] Oviedo S., Contreras I., Quirós C., Giménez M., Conget I., Vehi J. (2019). Risk-based postprandial hypoglycemia forecasting using supervised learning. Int. J. Med. Inform..

[63] Dave D., DeSalvo D.J., Haridas B., McKay S., Shenoy A., Koh C.J., Lawley M., Erraguntla M. (2021). Feature-based machine learning model for real-time hypoglycemia prediction. J. Diabetes Sci. Technol..

[64] Jin Y., Li F., Vimalananda V.G., Yu H. (2019). Automatic detection of hypoglycemic events from the electronic health record notes of diabetes patients: Empirical study. JMIR Med. Inform..

[65] Plis K., Bunescu R., Marling C., Shubrook J., Schwartz F. A machine learning approach to predicting blood glucose levels for diabetes management. Proceedings of the Workshops at the Twenty-Eighth AAAI Conference on Artificial Intelligence.

[66] Cescon M., Johansson R., Renard E. (2015). Subspace-based linear multi-step predictors in type 1 diabetes mellitus. Biomed. Signal Process. Control.

[67] Bertachi A., Biagi L., Contreras I., Luo N., Vehí J. Prediction of Blood Glucose Levels And Nocturnal Hypoglycemia Using Physiological Models and Artificial Neural Networks. Proceedings of the 3rd International Workshop on Knowledge Discovery in Healthcare Data, KDH@ IJCAI-ECAI 2018.

[68] Marling C., Bunescu R.C. The OhioT1DM Dataset For Blood Glucose Level Prediction. Proceedings of the 3rd International Workshop on Knowledge Discovery in Healthcare Data.

[69] Marling C., Bunescu R. The OhioT1DM dataset for blood glucose level prediction: Update 2020. Proceedings of the 5th International Workshop on Knowledge Discovery in Healthcare Data.

[70] Diabetes Research in Children Network (DirecNet) Study Group. Diabetes Research in Children Network (DirecNet). https://public.jaeb.org/direcnet.

[71] Lehmann E. (1998). Preliminary experience with the Internet release of AIDA—an interactive educational diabetes simulator. Comput. Methods Programs Biomed..

[72] Man C.D., Micheletto F., Lv D., Breton M., Kovatchev B., Cobelli C. (2014). The UVA/PADOVA type 1 diabetes simulator: New features. J. Diabetes Sci. Technol..

[73] Nemat H., Khadem H., Elliott J., Benaissa M. (2024). Physical Activity Integration in Blood Glucose Level Prediction: Different Levels of Data Fusion. IEEE J. Biomed. Health Inform..

[74] Nemat H., Khadem H., Eissa M.R., Elliott J., Benaissa M. (2022). Blood Glucose Level Prediction: Advanced Deep-Ensemble Learning Approach. IEEE J. Biomed. Health Inform..

[75] Ali J.B., Hamdi T., Fnaiech N., Di Costanzo V., Fnaiech F., Ginoux J.M. (2018). Continuous blood glucose level prediction of type 1 diabetes based on artificial neural network. Biocybern. Biomed. Eng..

[76] Hamdi T., Ali J.B., Di Costanzo V., Fnaiech F., Moreau E., Ginoux J.M. (2018). Accurate prediction of continuous blood glucose based on support vector regression and differential evolution algorithm. Biocybern. Biomed. Eng..

[77] D’Antoni F., Merone M., Piemonte V., Pozzilli P., Iannello G., Soda P. (2019). Early Experience in Forecasting Blood Glucose Levels Using a Delayed and Auto-Regressive Jump Neural Network. Proceedings of the 2019 IEEE 18th International Conference on Cognitive Informatics & Cognitive Computing (ICCI* CC).

[78] Alfian G., Syafrudin M., Anshari M., Benes F., Atmaji F.T.D., Fahrurrozi I., Hidayatullah A.F., Rhee J. (2020). Blood glucose prediction model for type 1 diabetes based on artificial neural network with time-domain features. Biocybern. Biomed. Eng..

[79] Dudukcu H.V., Taskiran M., Yildirim T. (2021). Blood glucose prediction with deep neural networks using weighted decision level fusion. Biocybern. Biomed. Eng..

[80] Zhu T., Li K., Chen J., Herrero P., Georgiou P. (2020). Dilated Recurrent Neural Networks for Glucose Forecasting in Type 1 Diabetes. J. Healthc. Inform. Res..

[81] Güemes A., Cappon G., Hernandez B., Reddy M., Oliver N., Georgiou P., Herrero P. (2019). Predicting quality of overnight glycaemic control in type 1 diabetes using binary classifiers. IEEE J. Biomed. Health Inform..

[82] Lubasinski N., Thabit H., Nutter P.W., Harper S. (2024). Blood Glucose Prediction from Nutrition Analytics in Type 1 Diabetes: A Review. Nutrients.

[83] Zecchin C., Facchinetti A., Sparacino G., Cobelli C. (2016). How much is short-term glucose prediction in type 1 diabetes improved by adding insulin delivery and meal content information to CGM data? A proof-of-concept study. J. Diabetes Sci. Technol..

[84] Hameed H., Kleinberg S. Comparing machine learning techniques for blood glucose forecasting using free-living and patient generated data. Proceedings of the Machine Learning for Healthcare Conference.

[85] Jeon J., Leimbigler P.J., Baruah G., Li M.H., Fossat Y., Whitehead A.J. (2020). Predicting glycaemia in type 1 diabetes patients: Experiments in feature engineering and data imputation. J. Healthc. Inform. Res..

[86] Ngo P., Lu J., Pham H., Nguyen H., Phung D. (2021). Predicting blood glucose dynamics with machine learning: A review. IEEE Rev. Biomed. Eng..

[87] Kovatchev B. (2019). Automated closed-loop control of diabetes: The artificial pancreas. Bioeng. Transl. Med..

[88] Xie J., Wang Q. (2020). Benchmarking Machine Learning Algorithms on Blood Glucose Prediction for Type I Diabetes in Comparison with Classical Time-Series Models. IEEE Trans. Biomed. Eng..

[89] Clarke W.L. (2005). The original Clarke error grid analysis (EGA). Diabetes Technol. Ther..

[90] Parkes J.L., Slatin S.L., Pardo S., Ginsberg B.H. (2000). A new consensus error grid to evaluate the clinical significance of inaccuracies in the measurement of blood glucose. Diabetes Care.

[91] Klonoff D.C., Lias C., Vigersky R., Clarke W., Parkes J.L., Sacks D.B., Kirkman M.S., Kovatchev B., Panel E.G. (2014). The surveillance error grid. J. Diabetes Sci. Technol..

[92] Li K., Daniels J., Liu C., Herrero P., Georgiou P. (2019). Convolutional recurrent neural networks for glucose prediction. IEEE J. Biomed. Health Inform..

[93] Zhu T., Li K., Herrero P., Georgiou P. (2022). Personalized blood glucose prediction for type 1 diabetes using evidential deep learning and meta-learning. IEEE Trans. Biomed. Eng..

[94] Khadem H., Nemat H., Elliott J., Benaissa M. (2023). Blood Glucose Level Time Series Forecasting: Nested Deep Ensemble Learning Lag Fusion. Bioengineering.

[95] Wilcoxon F. (1945). Individual Comparisons by Ranking Methods. Biom. Bull..

[96] Friedman M. (1940). A comparison of alternative tests of significance for the problem of m rankings. Ann. Math. Stat..

[97] Fisher R. (1955). Statistical methods and scientific induction. J. R. Stat. Soc. Ser. B (Methodol.).

[98] Nemenyi P.B. (1963). Distribution-Free Multiple Comparisons.

[99] Holm S. (1979). A simple sequentially rejective multiple test procedure. Scand. J. Stat..

[100] Demšar J. (2006). Statistical comparisons of classifiers over multiple data sets. J. Mach. Learn. Res..

[101] Nemat H., Khadem H., Elliott J., Benaissa M. (2023). Causality analysis in type 1 diabetes mellitus with application to blood glucose level prediction. Comput. Biol. Med..

[102] Ribeiro M.T., Singh S., Guestrin C. Why Should I Trust You?: Explaining the Predictions of Any Classifier. Proceedings of the 22nd ACM SIGKDD International Conference on Knowledge Discovery and Data Mining.

[103] Lundberg S.M., Lee S.I. A Unified Approach to Interpreting Model Predictions. Proceedings of the Advances in Neural Information Processing Systems.

[104] Che Z., Purushotham S., Cho K., Sontag D., Liu Y. (2018). Recurrent neural networks for multivariate time series with missing values. Sci. Rep..

[105] Shukla S.N., Marlin B.M. (2019). Interpolation-prediction networks for irregularly sampled time series. arXiv.

[106] Bach K., Bunescu R., Marling C., Wiratunga N. Preface the 5th international workshop on knowledge discovery in healthcare data (KDH). Proceedings of the 5th Annual Workshop on Knowledge Discovery in Healthcare Data.

[107] Svozil D., Kvasnicka V., Pospichal J. (1997). Introduction to multi-layer feed-forward neural networks. Chemom. Intell. Lab. Syst..

[108] Hubel D.H., Wiesel T.N. (1968). Receptive fields and functional architecture of monkey striate cortex. J. Physiol..

[109] Rumelhart D.E., Hinton G.E., Williams R.J. (1986). Learning representations by back-propagating errors. Nature.

[110] Vaswani A., Shazeer N., Parmar N., Uszkoreit J., Jones L., Gomez A.N., Kaiser L., Polosukhin I. Attention Is All You Need. Proceedings of the Advances in Neural Information Processing Systems.

[111] Brownlee J. (2016). Deep Learning with Python: Develop Deep Learning Models on Theano and TensorFlow Using Keras.

[112] Krenker A., Bešter J., Kos A. (2011). Introduction to the artificial neural networks. Artificial Neural Networks: Methodological Advances and Biomedical Applications.

[113] Hochreiter S., Schmidhuber J. (1997). Long short-term memory. Neural Comput..

[114] Cho K., Van Merriënboer B., Gulcehre C., Bahdanau D., Bougares F., Schwenk H., Bengio Y. (2014). Learning phrase representations using RNN encoder-decoder for statistical machine translation. arXiv.

[115] Zhou H., Zhang S., Peng J., Zhang S., Li J., Xiong H., Zhang W. Informer: Beyond Efficient Transformer for Long Sequence Time-Series Forecasting. Proceedings of the AAAI Conference on Artificial Intelligence.

[116] Lim B., Arik S.O., Loeff N., Pfister T. (2021). Temporal Fusion Transformers for Interpretable Multi-horizon Time Series Forecasting. Int. J. Forecast..

[117] Zhu T., Yao X., Li K., Herrero P., Georgiou P. Blood glucose prediction for type 1 diabetes using generative adversarial networks. Proceedings of the 5th Annual Workshop on Knowledge Discovery in Healthcare Data.

[118] De Bois M., El Yacoubi M.A., Ammi M. (2021). Adversarial multi-source transfer learning in healthcare: Application to glucose prediction for diabetic people. Comput. Methods Programs Biomed..

[119] Khadem H., Nemat H., Elliott J., Benaissa M. (2026). Personalised Blood Glucose Time Series Forecasting in Type 1 Diabetes: Deep Collaborative Adversarial Learning. J. Pers. Med..

[120] Zhang M., Flores K.B., Tran H.T. (2021). Deep learning and regression approaches to forecasting blood glucose levels for type 1 diabetes. Biomed. Signal Process. Control.

[121] Zhu T., Kuang L., Li K., Zeng J., Herrero P., Georgiou P. (2021). Blood glucose prediction in type 1 diabetes using deep learning on the edge. Proceedings of the 2021 IEEE International Symposium on Circuits and Systems (ISCAS).

[122] Mirshekarian S., Shen H., Bunescu R., Marling C. (2019). LSTM and Neural Attention Models for Blood Glucose Prediction: Comparative Experiments on Real and Synthetic Data. Proceedings of the 2019 41st Annual International Conference of the IEEE Engineering in Medicine and Biology Society (EMBC).

[123] Lee S.M., Kim D.Y., Woo J. (2023). Glucose Transformer: Forecasting Glucose Level and Events of Hyperglycemia and Hypoglycemia. IEEE J. Biomed. Health Inform..

[124] Alkanhel R.I., Saleh H., Elaraby A., Alharbi S., Elmannai H., Alaklabi S., Alsamhi S.H., Mostafa S. (2024). Hybrid CNN-GRU Model for Real-Time Blood Glucose Forecasting: Enhancing IoT-Based Diabetes Management with AI. Sensors.

[125] Manchanda E., Zeng J., Lo C.H. (2025). Data-Efficiency with Comparable Accuracy: Personalized LSTM Neural Network Training for Blood Glucose Prediction in Type 1 Diabetes Management. Diabetology.

[126] Daniels J., Herrero P., Georgiou P. (2021). A multitask learning approach to personalized blood glucose prediction. IEEE J. Biomed. Health Inform..

[127] Zhuang F., Qi Z., Duan K., Xi D., Zhu Y., Zhu H., Xiong H., He Q. (2020). A comprehensive survey on transfer learning. Proc. IEEE.

[128] Bhimireddy A., Sinha P., Oluwalade B., Gichoya J.W., Purkayastha S. (2020). Blood glucose level prediction as time-series modeling using sequence-to-sequence neural networks. CEUR Workshop Proceedings.

[129] Shuvo M.M.H., Islam S.K. (2023). Deep Multitask Learning by Stacked Long Short-Term Memory for Predicting Personalized Blood Glucose Concentration. IEEE J. Biomed. Health Inform..

[130] Sagi O., Rokach L. (2018). Ensemble learning: A survey. Wiley Interdiscip. Rev. Data Min. Knowl. Discov..

[131] Wadghiri M., Idri A., El Idrissi T., Hakkoum H. (2022). Ensemble blood glucose prediction in diabetes mellitus: A review. Comput. Biol. Med..

[132] Breiman L. (1996). Bagging predictors. Mach. Learn..

[133] Freund Y., Schapire R., Abe N. (1999). A short introduction to boosting. J.-Jpn. Soc. Artif. Intell..

[134] Breiman L. (1996). Stacked regressions. Mach. Learn..

[135] Martinsson J., Schliep A., Eliasson B., Meijner C., Persson S., Mogren O. Automatic blood glucose prediction with confidence using recurrent neural networks. Proceedings of the 3rd International Workshop on Knowledge Discovery in Healthcare Data, KDH@ IJCAI-ECAI 2018.

[136] Midroni C., Leimbigler P.J., Baruah G., Kolla M., Whitehead A.J., Fossat Y. (2018). Predicting glycemia in type 1 diabetes patients: Experiments with XGBoost. Heart.

[137] Saiti K., Macaš M., Lhotská L., Štechová K., Pithová P. (2020). Ensemble methods in combination with compartment models for blood glucose level prediction in type 1 diabetes mellitus. Comput. Methods Programs Biomed..

[138] Nemat H., Khadem H., Elliott J., Benaissa M. Data fusion of activity and CGM for predicting blood glucose levels. Proceedings of the 5th Annual Workshop on Knowledge Discovery in Healthcare Data.

[139] Khadem H., Nemat H., Elliott J., Benaissa M. Multi-lag stacking for blood glucose level prediction. Proceedings of the 5th Annual Workshop on Knowledge Discovery in Healthcare Data.

[140] Langarica S., Rodriguez-Fernandez M., Nunez F., Doyle F.J. (2023). A meta-learning approach to personalized blood glucose prediction in type 1 diabetes. Control Eng. Pract..

[141] Pearl J. (2000). Models, Reasoning and Inference.

[142] Xu G., Duong T.D., Li Q., Liu S., Wang X. (2020). Causality learning: A new perspective for interpretable machine learning. arXiv.

[143] Yao L., Chu Z., Li S., Li Y., Gao J., Zhang A. (2021). A survey on causal inference. ACM Trans. Knowl. Discov. Data (TKDD).

[144] Eichler M. (2013). Causal inference with multiple time series: Principles and problems. Philos. Trans. R. Soc. A Math. Phys. Eng. Sci..

[145] Siggiridou E., Koutlis C., Tsimpiris A., Kugiumtzis D. (2019). Evaluation of Granger causality measures for constructing networks from multivariate time series. Entropy.

[146] Kassani P.H., Xiao L., Zhang G., Stephen J.M., Wilson T.W., Calhoun V.D., Wang Y.P. (2020). Causality-Based Feature Fusion for Brain Neuro-Developmental Analysis. IEEE Trans. Med. Imaging.

[147] Javier P.J.E.A., Liponhay M.P., Dajac C.V.G., Monterola C.P. (2022). Causal network inference in a dam system and its implications on feature selection for machine learning forecasting. Phys. A Stat. Mech. Its Appl..

[148] Hmamouche Y., Przymus P., Casali A., Lakhal L. (2017). GFSM: A Feature Selection Method for Improving Time Series Forecasting. Int. J. Adv. Syst. Meas..

[149] Hmamouche Y., Casali A., Lakhal L. A causality based feature selection approach for multivariate time series forecasting. Proceedings of the DBKDA 2017, The Ninth International Conference on Advances in Databases, Knowledge, and Data Applications.

[150] Zhu T., Li K., Herrero P., Chen J., Georgiou P. A Deep Learning Algorithm for Personalized Blood Glucose Prediction. Proceedings of the 3rd International Workshop on Knowledge Discovery in Healthcare Data, KDH@ IJCAI-ECAI 2018.

[151] Van Den Oord A., Dieleman S., Zen H., Simonyan K., Vinyals O., Graves A., Kalchbrenner N., Senior A., Kavukcuoglu K. (2016). Wavenet: A generative model for raw audio. arXiv.

[152] He M., Gu W., Kong Y., Zhang L., Spanos C.J., Mosalam K.M. (2019). CausalBG: Causal recurrent neural network for the blood glucose inference with IoT platform. IEEE Internet Things J..

[153] Sugihara G., May R., Ye H., Hsieh C.h., Deyle E., Fogarty M., Munch S. (2012). Detecting causality in complex ecosystems. Science.

[154] Ye H., Deyle E.R., Gilarranz L.J., Sugihara G. (2015). Distinguishing time-delayed causal interactions using convergent cross mapping. Sci. Rep..

[155] Freiburghaus J., Rizzotti-Kaddouri A., Albertetti F. A deep learning approach for blood glucose prediction of type 1 diabetes. Proceedings of the 5th Annual Workshop on Knowledge Discovery in Healthcare Data.

[156] Rubin-Falcone H., Fox I., Wiens J. Deep Residual Time-Series Forecasting: Application to Blood Glucose Prediction. Proceedings of the 5th Annual Workshop on Knowledge Discovery in Healthcare Data.

[157] Hameed H., Kleinberg S. Investigating potentials and pitfalls of knowledge distillation across datasets for blood glucose forecasting. Proceedings of the 5th Annual Workshop on Knowledge Discovery in Healthcare Data.

[158] Yang T., Wu R., Tao R., Wen S., Ma N., Zhao Y., Yu X., Li H. Multi-Scale Long Short-Term Memory Network with Multi-Lag Structure for Blood Glucose Prediction. Proceedings of the 5th Annual Workshop on Knowledge Discovery in Healthcare Data.

[159] Bevan R., Coenen F. Experiments in non-personalized future blood glucose level prediction. Proceedings of the 5th Annual Workshop on Knowledge Discovery in Healthcare Data.

[160] Sun X., Rashid M.M., Sevil M., Hobbs N., Brandt R., Askari M.R., Shahidehpour A., Cinar A. Prediction of Blood Glucose Levels for People with Type 1 Diabetes using Latent-Variable-based Model. Proceedings of the 5th Annual Workshop on Knowledge Discovery in Healthcare Data.

[161] Daniels J., Herrero P., Georgiou P. Personalised Glucose Prediction via Deep Multitask Networks. Proceedings of the 5th Annual Workshop on Knowledge Discovery in Healthcare Data.

[162] Rodríguez-Rodríguez I., Rodríguez J.V., Molina-García-Pardo J.M., Zamora-Izquierdo M.Á., Martínez-Inglés M.T.M.I.I. (2020). A Comparison of Different Models of Glycemia Dynamics for Improved Type 1 Diabetes Mellitus Management with Advanced Intelligent Analysis in an Internet of Things Context. Appl. Sci..

[163] Nemat H., Khadem H., Elliott J., Benaissa M. (2024). Data-driven blood glucose level prediction in type 1 diabetes: A comprehensive comparative analysis. Sci. Rep..

